# Phase I dose escalation study of sorafenib plus S-1 for advanced solid tumors

**DOI:** 10.1038/s41598-021-84279-6

**Published:** 2021-03-01

**Authors:** Hui-Jen Tsai, Her-Shyong Shiah, Jang-Yang Chang, Wu-Chou Su, Nai-Jung Chiang, Li-Tzong Chen

**Affiliations:** 1 National Institute of Cancer Research, National Health Research Institutes, Tainan, Taiwan; 2 Department of Oncology, National Cheng Kung University Hospital, College of Medicine, National Cheng Kung University, 1F No 367, Sheng-Li Road, Tainan, 70456 Taiwan; 3 Division of Hematology/Oncology, Department of Internal Medicine, Kaohsiung Medical University Hospital, Kaohsiung, Taiwan; 4 Division of Hematology and Oncology, Department of Internal Medicine, Taipei Tzu Chi Hospital, Buddhist Tzu Chi Medical Foundation, New Taipei, Taiwan; 5 Graduate Institute of Cancer Biology and Drug Discovery, Taipei Medical University, Taipei, Taiwan; 6 Institute of Biotechnology and Pharmaceutical Research, National Health Research Institutes, Zhunan, Taiwan; 7 Institute of Molecular Medicine, National Cheng Kung University, Tainan, Taiwan

**Keywords:** Targeted therapies, Chemotherapy

## Abstract

S-1, an oral pyrimidine fluoride-derived agent, is effective against various cancers. Sorafenib, an oral multikinase inhibitor, was found to prolong the survival of various cancers and enhance the cytotoxicity of chemotherapeutic agents. We conducted a phase I dose escalation study to determine dose-limiting toxicity (DLT) and maximal tolerated dose (MTD) of S-1 when combined with sorafenib for refractory solid tumors. Eligible patients received escalating doses (30, 35, and 40 mg/m^2^ bid) of S-1 Day 1 (D1)–D14 and continuous sorafenib 400 mg bid from cycle 1 D8 every 21 days in a standard 3 + 3 study design. Primary endpoint was MTD. Thirteen patients were enrolled between May 2010 and Feb 2012. DLT developed in two (one grade 3 erythema and one prolonged grade 2 hand-foot-skin reaction) of the 6 patients at 35 mg/m^2^ dose level. One pancreatic neuroendocrine tumor (pNET) patient achieved a durable partial response (27.9 months). Four colon cancer patients had stable disease and 3 of them had progression-free survival greater than 6 months. This study determined the recommended (MTD) S-1 dose of 30 mg/m^2^ bid for this regimen. This result warrants further phase II studies for advanced pNET and colon cancer to evaluate the efficacy of this combination.

## Introduction

Vascular endothelial growth factor (VEGF) is involved in angiogenesis and vascular permeability in cancers. Furthermore, it affects the function of immune cells in the tumor microenvironment and can promote the growth, survival, migration, and invasion of cancer cells^1^. Agents targeting VEGF, including VEGF antibody, bevacizumab, and VEGF receptor tyrosine kinase inhibitors (e.g. sunitinib and sorafenib), have either been used alone or in combination with chemotherapy to treat various cancers^2–4^. Sorafenib, a novel bi-aryl urea, is a potent inhibitor of Raf kinase and VEGF receptor-2 (VEGFR2). In an in vitro kinase assay, sorafenib had an inhibitory effect on multiple kinases, including c-Raf, wild type B-Raf, mutant B-Raf, and VEGFR-2, etc^5^. Its antitumor effect has also been confirmed in a variety of human cancer cell lines in vitro and in vivo^6^. The dosage and toxicity of sorafenib have been evaluated in phase I studies for cancer patients^7,8^. Sorafenib (400 mg twice daily) was approved by the Food and Drug Administration in the US for the treatment of advanced renal cell carcinoma in Dec 2005, unresectable hepatocellular carcinoma in Nov 2007, and progressive advanced differentiated thyroid cancer refractory to radioiodine treatment in Nov 2013^9^. To further improve the antitumor effect of antiangiogenesis agents, several strategies have been proposed, including a combination of antiangiogenesis agents with conventional cytotoxic chemotherapy. This approach has been supported by several recently published randomized studies, which showed that adding bevacizumab to conventional cytotoxic agents improves treatment results in colorectal cancer patients. However, the mechanisms underlying this synergistic effect between anti-angiogenetic and chemotherapeutic agents remain elusive^2^.

S-1 is a new oral pyrimidine fluoride-derived anticancer agent that consists of tegafur [FT,5-fluoro-1-(tetrahydro-2-furanyl)-2,4(1H,3H)pyrimidinedione] and 2 classes of modulators, gimeracil (CDHP, [5-chloro-2,4-dihydroxypyridine]) and oteracil potassium (Oxo, [Monopotassium1,2,3,4-tetrahydro-2,4-dioxo- 1,3,5-triazine-6-carboxylate]). S-1 was developed to enhance the clinical advantage of oral fluoropyrimidine and ameliorate the disadvantage of gastrointestinal toxicity^10^. The pharmacology, pharmacokinetics (PK), and toxicology of S-1 have been studied in a variety of animal models^11–13^ and its efficacy and tolerability have been shown in advanced cancers of digestive system in clinical trials^14–16^. S-1 doses up to 80 mg/m^2^/day can be used as a single therapeutic agent or 35 to 60 mg/m^2^/day when combined with other cytotoxic agents in phase I/II studies. The VEGF receptor is one of the sorafenib targets and it plays a critical role in neovascularization^17^. In a preclinical xenograft mouse model, sorafenib’s ability to enhance the cytotoxicity of chemotherapeutic agents was revealed^18^. In addition to its anti-proliferative activity, the anti-angiogenetic effect of sorafenib is expected to improve the delivery of S-1 to its target site. Moreover, elevated circulating endothelial cells (CECs) and circulating endothelial progenitors (CEPs) were noted in cancer patients^19^. These two markers were shown to play important roles in neovascularization and tumor growth and to be surrogate markers for antiangiogenesis drugs in cancers^20^. Based on the predicted synergistic antitumor effect of both drugs, we conducted a phase I dose escalation study of sorafenib 400 mg twice daily plus S-1 30–40 mg/m^2^ daily for the treatment of advanced solid tumors. The primary endpoint was to determine the maximal tolerated dose (MTD) of S-1. The MTD will be considered as recommended dose (RD) for further phase II study of S-1 combined with sorafenib 400 mg twice daily. Secondary endpoints were objective tumor response rate, disease control rate, duration of the response for the treatment of advanced solid tumors, and dynamic changes of CECs and CEPs by the treatment in these patients.

## Results

### Patient characteristics

A total of 13 patients were enrolled between May 2010 and Feb 2012. Baseline patient characteristics are listed in Table 1. All patients were evaluable for response and toxicities. Seven patients were men and 6 were women. The median age was 58 years old (39–63 years). Primary cancer types were colon cancer (4 patients; including one rectal cancer), pancreatic cancer (3 patients), and gastric cancer, gall bladder cancer, cholangiocarcinoma, thymic adenosquamous cell carcinoma, pancreatic neuroendocrine tumor (pNET), and ampulla Vater cancer (one patient each). Twelve patients were stage IV cases while the remaining case was stage III. The patient with stage III had locally advanced pancreatic cancer and was treated with gemcitabine-based concurrent chemoradiation therapy followed by gemcitabine alone initially. The patient then received chemotherapy with nanoplatin and gemcitabine as second-line therapy prior to enrolment in this trial due to progressive disease (PD) following second-line treatment. Eight patients received 3 or more lines of systemic treatment prior to enrolment.

**Table 1:** Patient demographics.

	Dosage(mg/m^2^)		
	30 (n = 7)	35 (n = 6)	Total (n = 13)
**Age**	60.3 ± 2.4	50.7 ± 7.0	55.8 ± 6.9
**Median age**	61	51	58
**Gender**			
Male	4	3	7 (54%)
Female	3	3	6 (46%)
**ECOG performance status**			
1	7	4	11 (85%)
0	0	2	2 (15%)
**HBV/HCV**			
No	6	4	10 (77%)
Yes	1	2	3 (23%)
**TNM stage**			
III	1	0	1 (8%)
IV	6	6	12 (92%)
**Primary cancer type**			
Colon cancer	4	0	4 (31%)
Pancreatic cancer	1	2	3 (31%)
Pancreatic NET	0	1	1(8%)
Thymic carcinoma	1	0	1 (8%)
Cholangiocarcinoma	0	1	1 (8%)
Gastric cancer	0	1	1 (8%)
Gall bladder cancer	0	1	1 (8%)
Ampulla Vater cancer	1	0	1 (8%)
**Previous lines of treatment**			
1	0	3	3 (23%)
2	1	1	2 (15%)
3	3	1	4 (31%)
> 3	3	1	4 (31%)

### Drug delivery and toxicities

**Figure 1:**
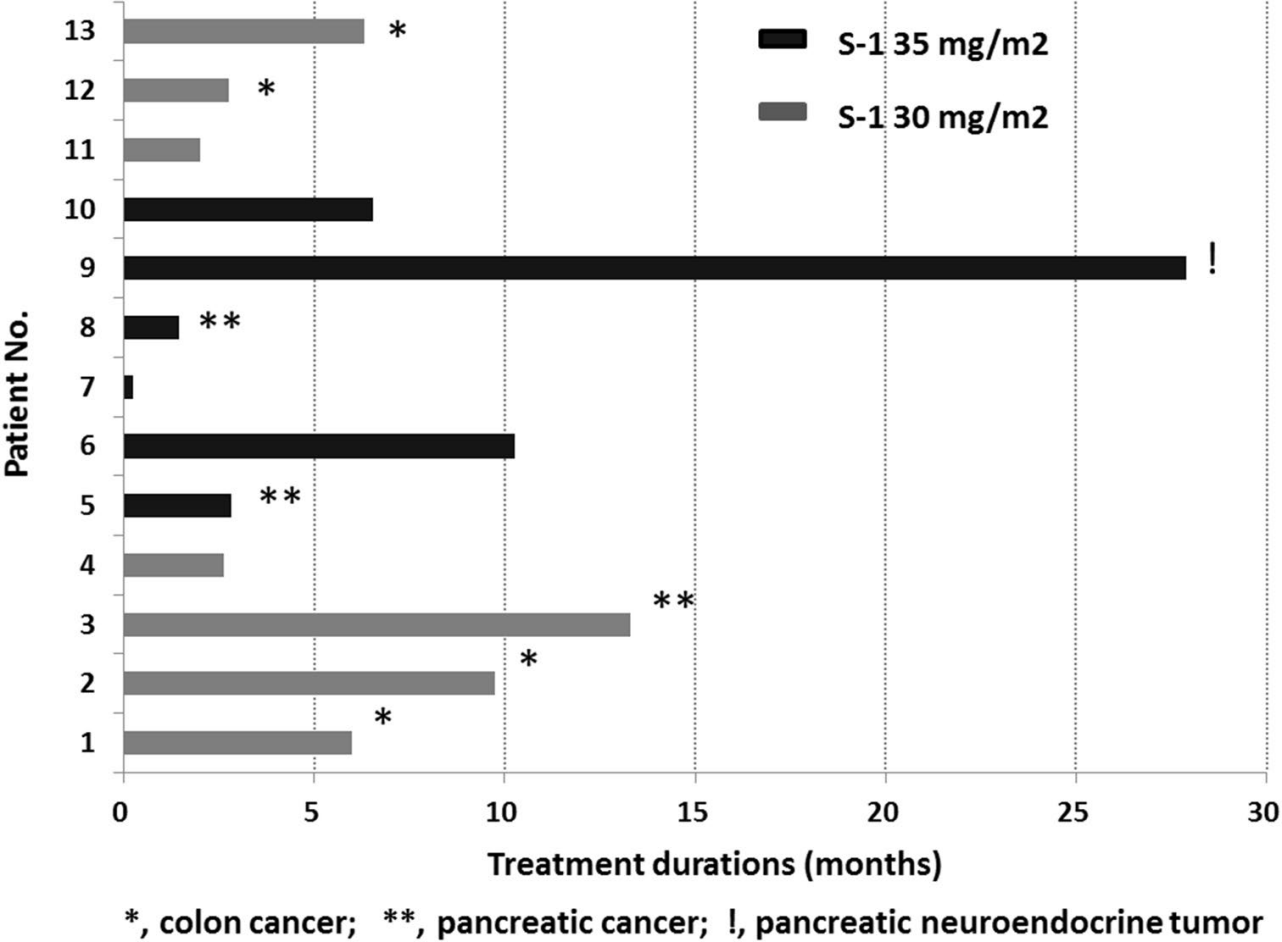
Treatment durations of patients in S-1 30 and 35 mg/m^2^ bid groups.

Of the first 3 patients administered 30 mg/m^2^, one was replaced for the dose escalation analysis as sorafenib dose was reduced after the first treatment cycle due to non-DLT grade 2 HFSR (recovery within 7 days). Therefore, a total of 4 patients were included in the first 30 mg/m^2^ S-1 cohort and DLT was not encountered in the 3 evaluable patients. One of the 3 patients treated with 35 mg/m^2^ of S-1 had DLT, which manifested as grade 3 skin erythema in the first 2 cycles of treatment. Another patient among the additional 3 patients treated with 35 mg/m^2^ of S-1 had prolonged (> 7 days) grade 2 HFSR developed in the first 2 cycles of treatment. Hence, 3 patients with S-1 30 mg/m^2^ dose were included; no DLT was noted in this cohort. Therefore, the MTD of S-1 was determined to be 30 mg/m^2^ bid. The median duration of treatment was 6.0 months (range 0.2–27.9 months). The treatment durations of all patients are shown in Fig. 1. Treatment-related toxicities in all patients are listed in Table 2. Overall, most common toxicities were mucositis (10/13, 76.9%) and HFSR (10/13, 76.9%) and most toxicities were grade I/II. Grade III toxicity, including fatigue, erythema, leukopenia, and hypokalemia, was only seen in one patient each. No patient died of drug-related toxicities.

**Table 2:** Treatment-related toxicities for all patients.

	Grade I	Grade II	Grade III	Grade IV	Total
	N (%)	N (%)	N (%)	N (%)	
**Dermatologic system**					
Mucositis	8 (61.5)	2 (15.4)	0	0	10 (76.9)
Hand-foot-skin reaction	3 (23.1)	7 (53.8)	0	0	10 (76.9)
Erythema	3 (23.1)	2 (15.4)	1 (7.7)	0	6 (46.2)
Hyperpigmentation	4 (30.8)	0	0	0	4 (30.8)
**Gastrointestinal system**					
GI upset	4 (30.8)	1 (7.7)	0	0	5 (38.5)
Nausea/vomiting	4 (30.8)	1 (7.7)	0	0	5 (38.5)
Diarrhea	2 (15.4)	0	0	0	2 (15.4)
**Constitutive symptom**					
Fatigue	7 (53.8)	0	1 (7.7)	0	8 (61.5)
Anorexia	5 (38.5)	0	0	0	5 (38.5)
**Hematologic side effect**					
Leukopenia	1 (7.7)	0	1 (7.7)	0	2 (15.4)
Anemia	3 (23.1)	2 (15.4)	0	0	5 (38.5)
Thrombocytopenia	2 (15.4)	0	0	0	2 (15.4)
**Others**					
Cough	2 (15.4)	0	0	0	2 (15.4)
Musculoskeletal pain	2 (15.4)	0	0	0	2 (15.4)
Hypertension	3 (23.1)	1 (7.7)	0	0	4 (30.8)
Alopecia	2 (15.4)	0	0	0	2 (15.4)
Weight loss	3 (23.1)	0	0	0	3 (23.1)
Bleeding	1 (7.7)	0	0	0	1 (7.7)
**Laboratory abnormality**					
AST/ALT elevation	3 (23.1)	1 (7.7)	0	0	4 (30.8)
Hyperbilirubinemia	3 (23.1)	1 (7.7)	0	0	4 (30.8)
ALK increase	3 (23.1)	1 (7.7)	0	0	4 (30.8)
GGT increase	1 (7.7)	1 (7.7)	0	0	2 (15.4)
Amylase increase	1 (7.7)	0	0	0	1 (7.7)
Proteinuria	3 (23.1)	0	0	0	3 (23.1)
Hypokalemia	2 (15.4)	0	1 (7.7)	0	3 (23.1)
Hypophosphatemia	1 (7.7)	0	0	0	1 (7.7)
Hypoalbuminemia	3 (23.1)	0	0	0	3 (23.1)

### Antitumor response

**Figure 2:**
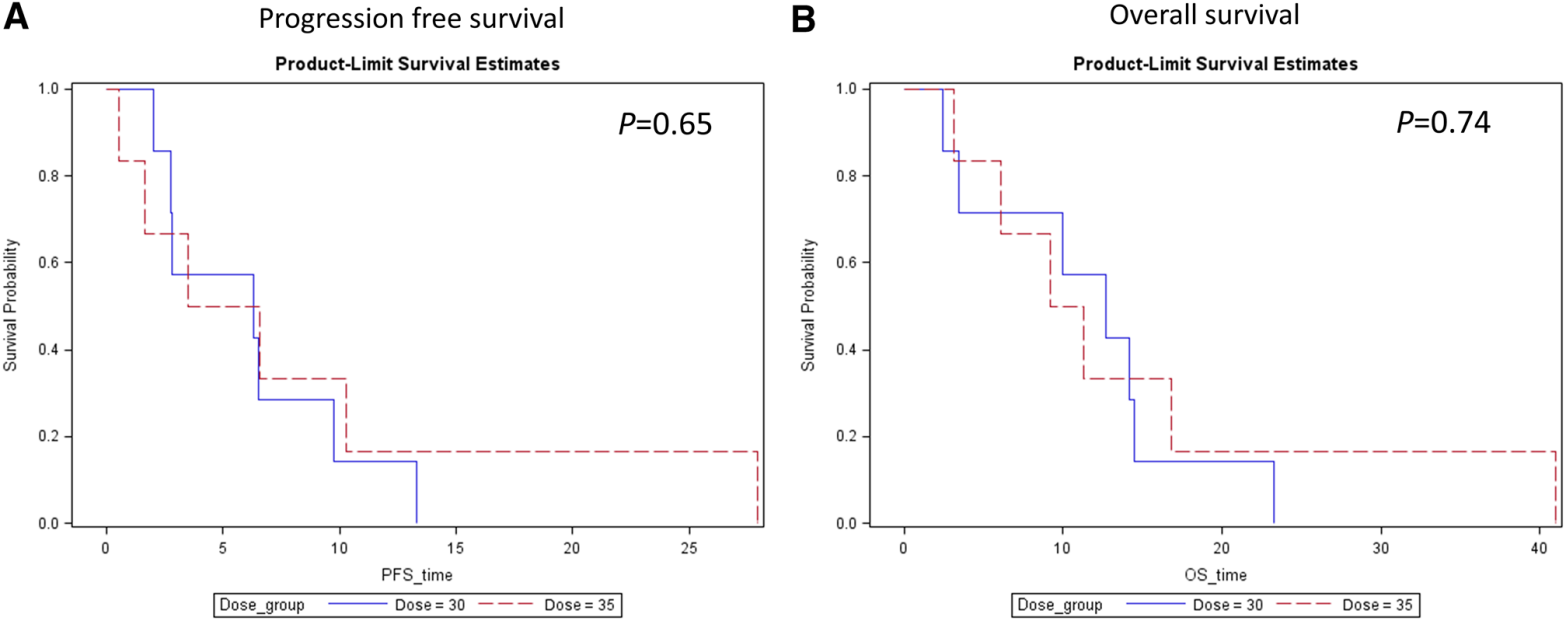
The progression free survival and overall survival of patients by S-1 dose level. (**A**) Progression free survival. (**B**) Overall survival.

The characteristics, best response, and progression free survival (PFS) for each patient are listed in Table 3. Tumor response was evaluated in all 13 patients. The best tumor response was a partial response (PR) in one patient, stable disease (SD) in 10 patients, and PD in 2 patients. Overall response rate and disease control rate were 7.7% and 84.6%, respectively. One patient with everolimus-refractory grade 2 pNET achieved PR with a PFS of 27.9 months. The 10 patients with SD had a median treatment duration of 6.2 months. Previous lines of treatment, dose level, and PFS of patients enrolled in this study are listed in Table 3. Median PFS was 6.3 months (95% confidence interval (CI), 2.8–9.8 months) and 5.1 months (95% CI, 1.7–10.3 months) for patients in S-1 30 mg/m^2^ and 35 mg/m^2^ groups, respectively. OS was 12.7 (95% CI 3.4–14.5 months) and 10.3 (6.1–16.8 months) months for patients in S-1 30 mg/m^2^ and 35 mg/m^2^ groups, respectively. The Kaplan–Meier curves of PFS and OS in S-1 30 mg/m^2^ and 35 mg/m^2^ groups are shown in Fig. 2.

**Table 3:** Characteristics and response of all patients.

Patient No.	Age	Gender	Dosage (mg/m^2^)	ECOG	T	N	M	TNM Stage	Cancer type	Previous lines of treatment	Best response	PFS (months)	OS* (Months)
000001	58	M	30	0	x	1	1	IV	Colon cancer	5	*SD*	6.3	14.2
000002	61	M	30	0	0	1	1	IV	Rectal cancer	8	*SD*	9.8	23.3
000003	59	F	30	0	4	1	0	III	Pancreatic cancer	2	*SD*	13.3	14.5
000004	57	M	30	0	x	1	1	IV	Thymic carcinoma	3	*SD*	2.8	3.4
000005	52	M	35	0	4	1	1	IV	Hepatocellular carcinoma	1	*SD*	3.5	6.1
000006	59	F	35	0	3	1	1	IVB	Cholangiocarcinoma	5	*SD*	10.3	16.8
000007	39	M	35	1	3	1	1	IV	Gastric cancer	2	*PD*	0.5	3.1
000008	50	F	35	0	x	1	1	IV	Pancreatic cancer	1	*PD*	1.7	9.2
000009	56	F	35	0	4	1	1	IV	Pancreatic NET	1	*PR*	27.9	41
000010	48	M	35	1	4	1	1	IV	Gall bladder cancer	3	*SD*	6.6	11.3
000011	63	M	30	0	3	1	1	IV	Ampulla of Vater cancer	3	*SD*	2.0	2.4
000012	63	F	30	0	4	1	1	IVA	Colon cancer	4	*SD*	2.8	10
000013	61	F	30	0	3	1	1	IV	Colon cancer	3	*SD*	6.5	12.7

*All patients were dead at the time of analysis.

### Changes of CECs and CEPs

**Figure 3:**
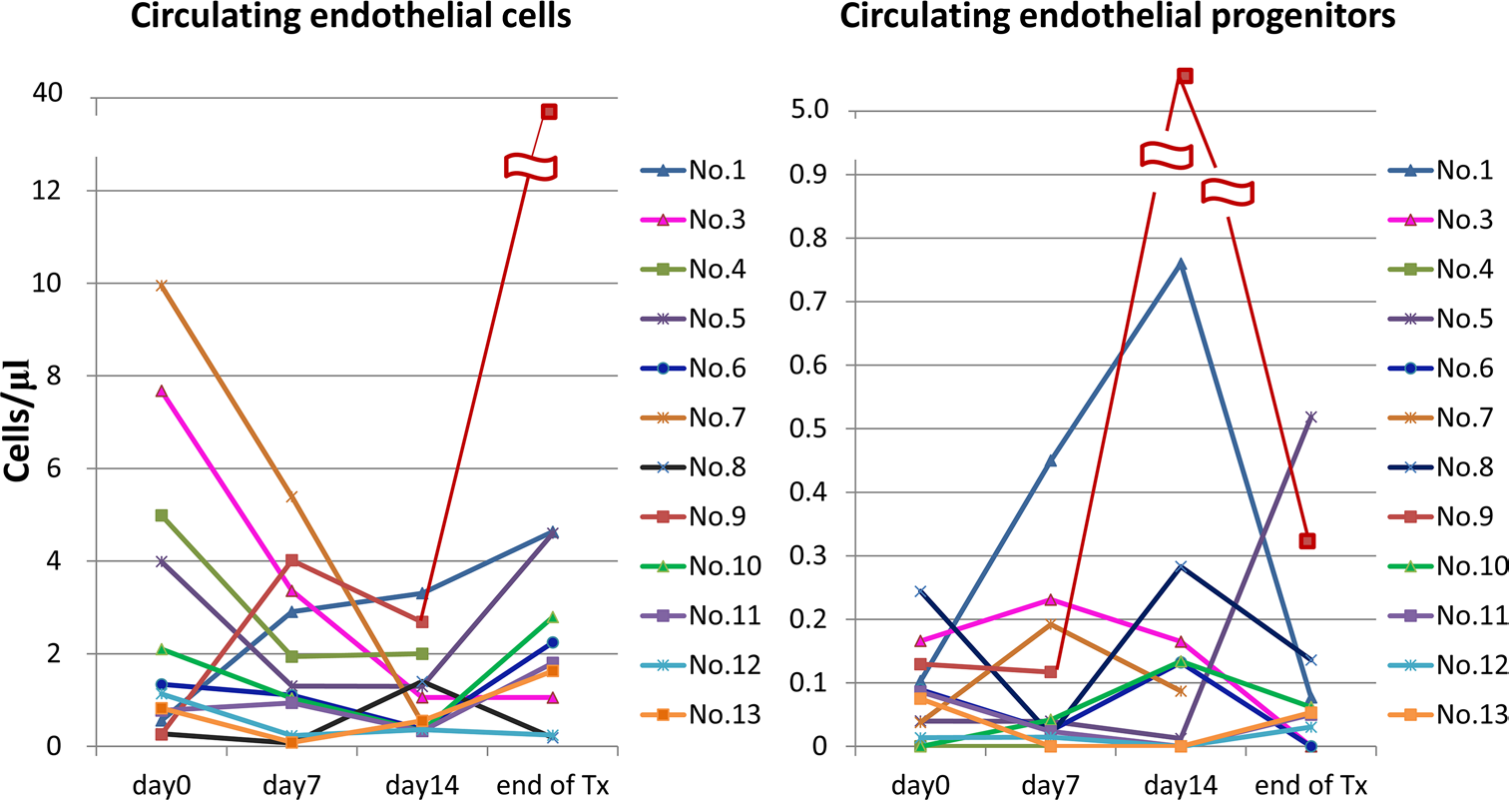
The dynamic changes of circulating endothelial cells (CECs) and circulating endothelial progenitors (CEPs) in 12 patients at baseline and after treatment. (**A**) The CECs of the 12 patients at D0, D7, D14 and at the end of treatment. (**B**) The CEPs of the 12 patients at D0, D7, D14 and at the end of treatment.

Because one patient who received reduced dose of sorafenib in S-1 30 mg/m^2^, this patient was not included for analysis of changes of CECs and CEPs in this study. The changes of CECs and CEPs from baseline (D0) to D7, D14 and end of treatment was presented in Fig. 3. The median CECs of the 12 patients at D0, D7, D14 and end of the treatment were 1.24, 1.20, 0.80, and 2.02 cells/μl, respectively. The median alive CECs of the 12 patients at D0, D7, D14 and end of the treatment were 0.90, 0.77, 0.34, and 1.19 cells/μl, respectively. The median CEPs of the 12 patients at D0, D7, D14 and end of the treatment were 0.08, 0.03, 0.11, and 0.06 cells/μl, respectively. The changes of CECs in the patients by S-1 (D7 vs D0, *P* = 0.11) or combination of S-1 and sorafenib (D14 vs D0, *P* = 0.18) were not significantly different. The viable CECs in the patients by S-1 were not significantly different (D7 vs D0, *P* = 0.15) but were decreased by combination of S-1 and sorafenib (D14 vs D0, *P* = 0.02). Furthermore, the viable CECs were significantly increased when the patients had disease progression (at the end of treatment vs D14, *P* = 0.02). The changes of CEPs in the patients were not significantly different either by S-1, combination of S-1 and sorafenib or at the time of disease progression.

## Discussion

We evaluated the safety and efficacy of combining S-1 and sorafenib in Taiwanese patients with advanced solid tumors. We found that 30 mg/m^2^ bid given on D1 to D14 of 3-week cycle is the optimal dose of S-1 for combination with sorafenib 400 mg bid starting from C1D8 continuously for treating advanced cancer patients.

Because S-1 was developed to enhance the clinical advantage of oral fluoropyrimidine and reduce the disadvantage of gastrointestinal toxicities, many phase II trials have been conducted to evaluate its response and toxicities in various cancers^15,21,22^. S-1 was shown to be effective for advanced gastric cancer. To add, it was found to be non-inferior to infusional fluorouracil and had a significant safety advantage^23^. Adjuvant S-1 was shown to improve PFS and OS in stage II or III patients treated via gastrectomy with extended (D2) lymph-node dissection compared to patients receiving only surgery in Japan^24^, where it is used as a standard treatment. The addition of sequential paclitaxel did not improve disease-free survival relative to treatment with S-1 alone. S-1 has also demonstrated non-inferiority to infusional fluorouracil for metastatic colorectal cancers or in adjuvant settings^25–29^. Van den Brande et al. investigated the administration of oral S-1 with 35–40 mg/m^2^ bid for 28 days with a rest period of one week as first-line treatment for advanced or metastatic colorectal cancer patients. In 37 evaluable patients, overall response and disease control rate were 24% and 70%, respectively^25^. Zhang et al. demonstrated the effectiveness of combining oxaliplatin 130 mg/m^2^ on D1 with oral S-1 40 mg/m^2^ bid from D1 to D14 every 3 weeks as first-line treatment for metastatic colorectal cancer patients. The overall response rate for 48 patients was 54%, the disease control rate was 90%, and the median time to tumor progression and OS were 8.5 and 27.2 months, respectively. The 2-year survival rate was 53% for intention to treat patients^26^. Muro et al. reported their phase III study results using irinotecan plus S-1 (IRIS) versus fluorouracil and folinic acid plus irinotecan (FOLFIRI) as a second-line treatment for metastatic colorectal cancer patients. For a total of 426 patients, median PFS was 5.1 months in the FOLFIRI group and 5.8 months in the IRIS group (hazard ratio (HR) 1.077, 95% CI 0.879–1.319, non-inferiority test *P* = 0.039). OS was 18.2 months in the FOLFIRI group and 19.5 months in the IRIS group (HR 0.909, 95% CI 0.699–1.181). Patients in the IRIS group had less grade 3/4 neutropenia but more diarrhea of all grades and grade 3 than patients in the FOLFIRI group. Altogether, these authors demonstrated the non-inferiority of IRIS to FOLFIRI as a second-line treatment for metastatic colorectal cancer^27^. The non-inferiority of S-1 and oxaliplatin (SOX) plus bevacizumab versus leucovorin, fluorouracil, and oxaliplatin (mFLOFOX6) plus bevacizumab as first-line treatment for metastatic colorectal cancer was also demonstrated by Yamada et al. Median PFS was 11.5 months (95% CI 10.7–13.2) in mFOLFOX6 plus bevacizumab group and 11.7 months in the SOX plus bevacizumab group (HR 1.04, 95% CI 0.86–1.24, non-inferiority *P* = 0.014)^28^. Furthermore, Yoshida et al. attempted to compare the efficacy of S-1 to tegafur-uracil plus leucovorin (UFT/LV) as adjuvant chemotherapy for stage III colon cancers. For 1518 patients, the 3-year disease-free survival rate was 75.5% and 72.5% in the S-1 and UFT/LV group, respectively. Compared to UFT/LV, HR for disease-free survival in S-1 group was 0.85 (95% CI 0.70–1.03), demonstrating the non-inferiority of S-1 (non-inferiority log-rank test, *P* < 0.001)^29^. In our study, all 4 colorectal patients received more than 2 lines of chemotherapy, consisting of oxaliplatin, or irinotecan combined with infusional 5FU and leucovorin. Three of these patients had a PFS greater than 6 months with S-1 and sorafenib. These results suggest the efficacy of S-1 for refractory colorectal cancer.

S-1 was further tested on other cancers of the digestive system, such as hepatocellular carcinoma and pancreatic cancer^16,30^. In GEST study, median PFS for patients treated with gemcitabine, S-1, and gemcitabine plus S-1 (GS) were 4.1, 3.8, and 5.7 months, respectively. Combination of gemcitabine and S-1 was shown to prolong PFS for advanced pancreatic cancer patients in Japan and Taiwan. Median OS of patients treated with gemcitabine, S-1, and GS were 8.8, 9.7, and 10.1 months, respectively. Hence, S-1 was demonstrated to be non-inferior to gemcitabine in OS of advanced pancreatic cancer patients^16^. In the current study, one pancreatic cancer patient, who failed to respond to a gemcitabine-based treatment, had disease control for 13.3 months when treated with S-1 and sorafenib.

**Figure 4:**
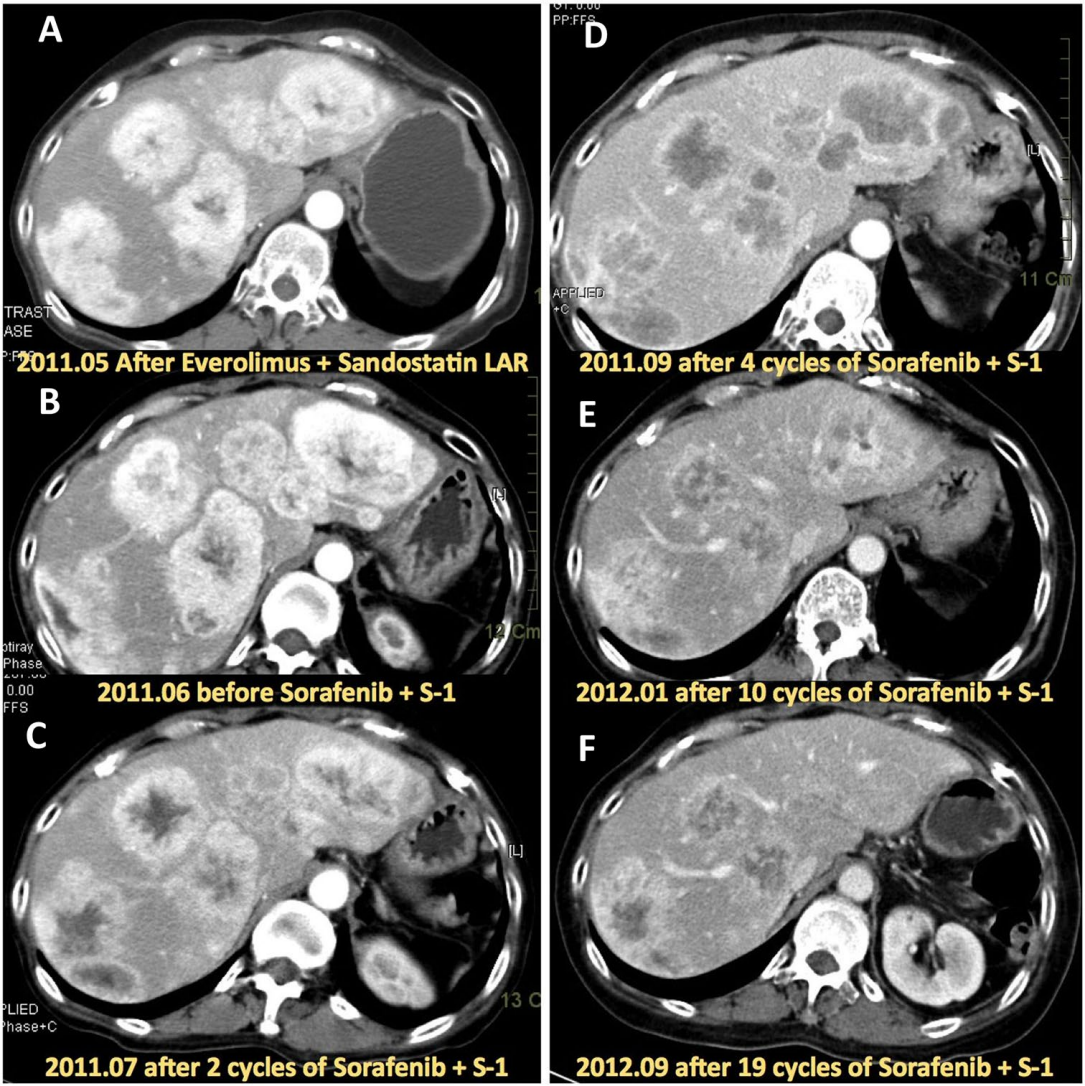
Serial CT images of a pNET patient enrolled in this study. (**A**) Hypervascular lesion of liver metastases; this patient failed to respond toeverolimus + Sandostatin LAR. (**B**) Rapid enlargement of liver metastases prior to treatment with sorafenib + S-1. (**C**)Regression of hypervascular lesion of liver metastases after 2 cycles of sorafenib + S-1. (**D**) Further regression of hypervascular lesion of liver metastases after 4 cycles of sorafenib + S-1. (**E**) Shrinkage of liver metastases after 10 cycles of sorafenib + S-1. (**F**) Persistent shrinkage of liver metastases after 19 cycles of sorafenib + S-1.

The pNET patient achieved PR after treatment with sorafenib and S-1 and had a durable response lasting 27.9 months. This patient was administered everolimus and Octreotide (sandostatin LAR) as a first-line treatment but disease progression occurred 21 months later. In Jun 2011, the patient began receiving sorafenib 400 mg bid and S-1 35 mg/m^2^/bid. Regression of the hypervascular liver metastatic tumors was noted after 2 cycles of this regimen and further shrinkage of liver tumors could be seen after 10 and 19 cycles of regimen (Fig. 4). Our study had 2 cases with PD and although most of the other 9 patients had SD, 6 had time to tumor progression of more than 6 months. These patients had the following cancer types: colorectal cancer (3 patients), and pancreatic adenocarcinoma, cholangiocarcinoma, and gall bladder cancer (one patient each). All received more than 2 lines of treatment. This result suggested that the combination of sorafenib and S-1 may effectively control tumor progression of cancers of the digestive system.

Sorafenib is an oral multikinase inhibitor that has anti-proliferative and antiangiogenesis activity. Previously, sorafenib was proven to prolong PFS and OS of advanced hepatocellular carcinoma and renal cell carcinoma and PFS of differentiated thyroid cancer through phase III trials^31–33^. Adding sorafenib to the regimen with chemotherapeutic agents has been shown to cause synergistic effects in various cancers. Co-administration of sorafenib and S-1was found to be effective against several cancer types in vitro and in vivo. Nukatsuka et al. showed that combining sorafenib and S-1 resulted in a synergistic antitumor effect on breast and non-small cell lung cancer cells in nude mice xenografts^34^. Zhai et al. showed that sorafenib enhanced the efficacy of S-1 against hepatocellular carcinoma in vitro and in vivo by downregulating the transcription factor, E2F-1^35^. In clinical settings, Lee et al. published a phase I trial of sorafenib combined with S-1 for advanced hepatocellular carcinoma patients. They recommended that sorafenib 400 mg bid daily (D1-D21) and S-1 40 mg/m^2^ bid daily (D1-D14) every 3 weeks can be tolerated with modest clinical efficacy (PFS,3.9 months; OS, 10.4 months)^36^. Ooka et al. also recommended the S-1 64 mg/m2/day combined with sorafenib 800 mg/day as the MTD in advanced hepatocellular carcinoma by a phase I/II study^37^. In the current study, MTD of S-1 was 30 mg/m^2^ bid for our population, a dose lower than that recommended in the 2 phase I studies. This result suggests that the tolerable dose of S-1 should be lower in the Taiwanese population. By combining sorafenib with S-1, Lee et al. showed that toxicities were minimal, with impaired liver function (25%) as the most common grade 3/4 toxicities^36^. Our study also demonstrated that only minimal toxicity was induced by this regimen.

VEGF might be an important prognostic marker and a therapeutic consideration in NETs^38^. In preclinical studies, tyrosine kinase inhibitors (TKIs) targeting VEGF alone or in combination with a chemotherapeutic agent (S-1 and/or gemcitabine) have been shown to be effective for pNET in vivo^39,40^. Sunitinib, a multi-targeted TKI that displays anti-VEGFR2 effect was found to prolong the PFS of advanced pNET patients^3^. Combination of sorafenib and bevacizumab has also been shown to exert modest effects on advanced NETs, including pNET, in a phase II study^41^. In our study, the pNET patient had PR to sorafenib + S-1 with a response duration of 27.9 months. This result demonstrates the good response of pNET to this regimen and suggests the need for a further phase II study to evaluate the efficacy of this combination for pNET patients. Moreover, CECs and CEPs has been shown to represent the angiogenesis in cancers and as a surrogate marker for antiangiogenesis after cancer treatment^19,20^. Decreased CECs were noted after chemotherapy or combination of chemotherapy with anti-angiogenesis target agents and lower CECs after treatment were correlated with better response rate and survival^42,43^. However, sorafenib may increase the level of CECs^44^. The CEPs may increase after cytotoxic agents^45^. In our study, CECs and CEPs were not significantly changed by S-1 or combination of S-1 and sorafenib. Only alive CECs were significantly decreased by combination of S-1 and sorafenib and the alive CECs increased when disease progression developed. The result suggests the anti-angiogenetic effect of combination of S-1 and sorafenib. The result suggests that alive CECs as the surrogate marker for antiangiogenesis and the tumor control. However, further study is needed to verify the effect due to lower case number in our study.

In conclusion, through this phase I study, we revealed the MTD when S-1 30 mg/m^2^ bid D1-D14 and continuous sorafenib 400 mg bid daily from C1D8 every 3 weeks were administered to Taiwanese patients. Toxicities were minimal and tolerable with a PR to pNET and durable disease control for heavily treated patients with gastrointestinal tract cancers, particularly colon cancer. Phase II studies are required to further evaluate these disease entities.

## Patients and methods

### Patient selection

Patients histologically or cytologically proven to have metastatic or locally advanced malignant solid tumors and failed to respond to standard therapy were eligible to participate in this study. Eligible patients were ≥ 20 and ≤ 75 years old, with adequate performance status (ECOG 0–2) and normal hematologic (absolute neutrophil count ≥ 1500/μl, hemoglobin ≥ 9 g/dl, Platelet ≥ 100,000/μl), hepatic (total bilirubin < 1.5 times of upper limit of normal (ULN; ≤ 3 X ULN in patients treated by drainage of obstructive jaundice), ALT and AST ≤ 2.5 X ULN (≤ 5 X ULN for patients with liver involvement of cancer)), and renal (serum creatinine ≤ 1 X ULN) functions. Their life expectancy should also be ≥ 12 weeks. Patients who were pregnant, lactating, or presented severe comorbidities (e.g., infection, heart failure, myocardial infarction) within 12 months or known hypersensitivity to any drug component of the treatment regimen were ineligible. The study was approved by the institutional review board of the National Cheng Kung University Hospital, and all patients provided signed informed consent. In addition, this trial was performed in accordance with the guidelines and regulations of the National Cheng Kung University Hospital. This study was registered on ClinicalTrials.gov (NCT01128998) on May 24, 2010 (https://clinicaltrials.gov/ct2/show/NCT01128998?term=NCT01128998&draw=2&rank=1).

### Dosage and drug administration

S-1 (30–40 mg/m^2^ bid) was administered for 14 days every one cycle (21 days) and sorafenib (400 mg bid) was administered continuously to patients from Day (D) 8 of the first cycle. Treatment was continued until disease progression, unacceptable toxicities, death, or patient/investigator withdrawal from the study. The dose of sorafenib was fixed at 400 mg twice daily (800 mg/day) while starting dose of S-1 in dose level I cohort was 30 mg/m^2^ bid; this dose would be escalated by 5 mg/m^2^ increments between dose levels. If 2 or more dose-limiting toxicity (DLT) occurred at dose level I, then dose de-escalation was performed. The technique of dose escalation and de-escalation is listed in Supplement Table 1. Intra-patient dose escalation was not allowed. At least 3 patients were enrolled in each dose level. If none of the first 3 patients experienced DLT, then dose escalation proceeded to the next cohort of patients. If one of 3 patients developed DLT, the cohort was expanded to 6 patients. If 2 of 3 or ≥ 2 of 6 patients developed DLT at a certain dose level, dose escalation was withheld and the prior dose level was verified as MTD.

### Evaluation

Pretreatment evaluation procedures included physical examination, computed tomography (CT), or magnetic resonance imaging scan for measurable lesions. Follow-up imaging studies were performed after completion of 2 cycles of treatment to evaluate tumor response. Treatment response was assessed according to the Response Evaluation Criteria in Solid Tumors (RECIST) v1.1. Complete blood count and serum biochemistry were measured before each cycle of treatment and additional measurements were taken as needed during the treatment period. Toxicities were assessed according to National Cancer Institute Common Terminology Criteria for Adverse Events (NCI-CTCAE, version 4.0). DLT was defined as toxicity during the first and second cycles consisting of one or more of the following events, including un-manageable grade 3 or 4 non-hematological toxicity (except for nausea/vomiting/alopecia or non-clinically significant laboratory changes), grade 4 hematological toxicity > 7 days, grade 3–4 hematological toxicity associated with complications (e.g., febrile neutropenia or bleeding), and prolonged (> 21 days) delay of study drug administration at C3D1 due to persistent drug-related adverse events after previous cycle of treatment. The dose of sorafenib was modified or even discontinued in patients with severe hand-foot-skin reaction (HFSR) or hypertension within first 2 cycles of treatment, as described in Supplement Tables 2 and 3. CECs and CEPs were measured at baseline (pre-treatment), Day (D) 7, and D14 of cycle 1 and the end date of treatment.

### Measurement of CECs and CEPs

Fresh blood samples were stained for anti-CD45 (to exclude hematopoietic cells) (eBioscience, San Diego, CA), anti-CD31 (an endothelial marker) (BD Biosciences, San Jose, CA), anti-CD133 (a progenitor cell marker) (MiltenyiBiotec, Auburn, CA), anti-CD146 (an endothelial cell marker) (Millipore, Temecula, CA) and the apoptosis marker 7-aminoactinomycin D (7AAD) (Merck, Buenos Aires, S.A.). The red blood cells were lysed by red cell lysis buffer and CECs and CEPs were analyzed by FACS (BD Biosciences). CECs were defined as negative for CD45, positive for CD146 and CD31, and negative for the progenitor marker CD133. CEPs were depicted by expression of CD133.

### Statistical consideration

The phase I trial aimed to evaluate the MTD of S-1 in combination with a full dose of sorafenib in a conventional 3 + 3 study design. Primary endpoint was to determine the MTD of S-1. The MTD would be considered as the recommended dose (RD) for further phase II study. Secondary endpoints were objective tumor response rate, disease control rate, and duration of response. We estimated that the MTD of S-1 would be between 30 and 40 mg/m^2^ bid; this would be allowed by the testing of 3 dose levels. The number of enrolled patients was expected to range from 9 to 18. Progression-free survival (PFS) and overall survival (OS) were estimated by Kaplan–Meier survival curves and were evaluated statistically with log-rank tests. All analyses were performed with SAS 9.2 (Cary, NC, USA). The dynamic changes of CECs and CEPs in the patients were analyzed by Wilcoxon sign rank test. A p-value less than 0.05 was considered statistically significant.

## Supplementary Information

Supplementary Table 1.

Supplementary Table 2.

Supplementary Table 3.
