# Supplementary material for: Phase I dose escalation study of sorafenib plus S-1 for advanced solid tumors
Source: Sci Rep. 2021 Mar 1;11:4834. doi: 10.1038/s41598-021-84279-6 (PMC7921110; doi:10.1038/s41598-021-84279-6)
Supplement: Supplementary file 1 — Supplementary Table 1. [file 41598_2021_84279_MOESM1_ESM.doc]

**Phase I dose escalation study of sorafenib plus S-1 for advanced solid tumors**

Hui-Jen Tsai^1,2,3^, Her-Shyong Hsiah^4,5^, Jang-Yang Chang^2^, Wu-Chou Su^2^, Nai-Jung Chiang^1,2^, Li-Tzong Chen^1,2,6,7^

^1^National Institute of Cancer Research, National Health Research Institutes, Tainan, Taiwan

^2^Division of Hematology/Oncology, Department of Internal Medicine, National Cheng Kung University Hospital, College of Medicine, National Cheng Kung University, Tainan, Taiwan

^3^Division of Hematology/Oncology, Department of Internal Medicine, Kaohsiung Medical University Hospital, Kaohsiung, Taiwan

^4^Department of Hematology and Oncology, Taipei Medical University Hospital, Taipei, Taiwan

^5^Graduate Institute of Cancer Biology and drug Discovery, Taipei Medical University, Taipei, Taiwan

^6^Division of Gastroenterology, Department of Internal Medicine, Kaohsiung Medical University Hospital, Kaohsiung Medical University, Kaohsiung, Taiwan

^7^Institute of Molecular Medicine, National Cheng Kung University, Tainan, Taiwan

# Supplement Table 1. Dose escalation schedule.

| **Dose Escalation Schedule** | | |
| --- | --- | --- |
|  | **Dose** | |
| **Dose Level** | **Sorafenib**  **(mg/bid)** | **S-1**  **(mg/m^2^ bid)** |
| Level I | 400 | 30 |
| Level II | 400 | 35 |
| Level III | 400 | 40 |
| Level Ia* | 400 | 25 |
| Level Ib** | 400 | 20 |
| * If 2 or more patients had DLT at dose level I, the S-1 dose level of Ia (25 mg/m^2^) was tested.  ** If 2 or more patients had DLT at S-1 dose level Ia, the S-1 dose level of Ib (20 mg/m^2^) was tested. | | |
