# Supplementary material for: Phase I dose escalation study of sorafenib plus S-1 for advanced solid tumors
Source: Sci Rep. 2021 Mar 1;11:4834. doi: 10.1038/s41598-021-84279-6 (PMC7921110; doi:10.1038/s41598-021-84279-6)
Supplement: Supplementary file 2 — Supplementary Table 2. [file 41598_2021_84279_MOESM2_ESM.doc]

**Phase I dose escalation study of sorafenib plus S-1 for advanced solid tumors**

Hui-Jen Tsai^1,2,3^, Her-Shyong Hsiah^4,5^, Jang-Yang Chang^2^, Wu-Chou Su^2^, Nai-Jung Chiang^1,2^, Li-Tzong Chen^1,2,6,7^

^1^National Institute of Cancer Research, National Health Research Institutes, Tainan, Taiwan

^2^Division of Hematology/Oncology, Department of Internal Medicine, National Cheng Kung University Hospital, College of Medicine, National Cheng Kung University, Tainan, Taiwan

^3^Division of Hematology/Oncology, Department of Internal Medicine, Kaohsiung Medical University Hospital, Kaohsiung, Taiwan

^4^Department of Hematology and Oncology, Taipei Medical University Hospital, Taipei, Taiwan

^5^Graduate Institute of Cancer Biology and drug Discovery, Taipei Medical University, Taipei, Taiwan

^6^Division of Gastroenterology, Department of Internal Medicine, Kaohsiung Medical University Hospital, Kaohsiung Medical University, Kaohsiung, Taiwan

^7^Institute of Molecular Medicine, National Cheng Kung University, Tainan, Taiwan

Supplement Table 2. Dose Modification of Sorafenib in Hand-Foot Skin Reaction

| **Hand-Foot Skin Toxicity Grade** | **Occurrence** | **Dose Modification of Sorafenib** |
| --- | --- | --- |
| Grade 1:  Numbness, blunt, dysesthesia, pricking, painless swelling, erythema, but none of above interfering activity of daily life | Any time | Continuing sorafenib and considering local treatment for symptomatic relief |
| Grade 2:  Painfule erythema and hand/foot swelling, and/or inferfering activity of daily life | 1^st^ event lasting < 7 days | Continuing sorafenib and considering local treatment for symptomatic relief |
|  |  1^st^ event lasting > 7 days   2^nd^ event (duration of 1^st^ event < 7 days) | Discontinue until recovered to grade 0-1 and subsequent dose reduced to 400 mg QD |
|  |  2^nd^ event (duration of 1^st^ event > 7 days)   3^rd^ event | Discontinue until recovered to grade 0-1 and subsequent dose reduced to 200 mg QD |
|  | 4^th^ event | Discontinue |
| Grade 3:  Peeling, ulceration, blisters, severe pain, can not have activity of daily life | 1^st^ event | Discontinue until recovered to grade 0-1 and subsequent dose reduced to 400 mg QD |
|  | 2^nd^ event | Discontinue until recovered to grade 0-1 and subsequent dose reduced to 200 mg QD |
|  | 3^rd^ event | Discontinue |
