# Supplementary material for: Phase I dose escalation study of sorafenib plus S-1 for advanced solid tumors
Source: Sci Rep. 2021 Mar 1;11:4834. doi: 10.1038/s41598-021-84279-6 (PMC7921110; doi:10.1038/s41598-021-84279-6)
Supplement: Supplementary file 3 — Supplementary Table 3. [file 41598_2021_84279_MOESM3_ESM.doc]

**Phase I dose escalation study of sorafenib plus S-1 for advanced solid tumors**

Hui-Jen Tsai^1,2,3^, Her-Shyong Hsiah^4,5^, Jang-Yang Chang^2^, Wu-Chou Su^2^, Nai-Jung Chiang^1,2^, Li-Tzong Chen^1,2,6,7^

^1^National Institute of Cancer Research, National Health Research Institutes, Tainan, Taiwan

^2^Division of Hematology/Oncology, Department of Internal Medicine, National Cheng Kung University Hospital, College of Medicine, National Cheng Kung University, Tainan, Taiwan

^3^Division of Hematology/Oncology, Department of Internal Medicine, Kaohsiung Medical University Hospital, Kaohsiung, Taiwan

^4^Department of Hematology and Oncology, Taipei Medical University Hospital, Taipei, Taiwan

^5^Graduate Institute of Cancer Biology and drug Discovery, Taipei Medical University, Taipei, Taiwan

^6^Division of Gastroenterology, Department of Internal Medicine, Kaohsiung Medical University Hospital, Kaohsiung Medical University, Kaohsiung, Taiwan

^7^Institute of Molecular Medicine, National Cheng Kung University, Tainan, Taiwan

Supplement Table 3. Dose Modification of Sorafenib in Hypertension

| **Hypertension Toxicity Grade** | **Dose Interruption** | **Dose Modification of Sorafenib** |
| --- | --- | --- |
| Grade 1:  Asymptomatic and diastolic BP < 110 mmHg | Treat on time (Treat patient with anti-hypertensive agent) | No change |
| Grade 2:  Recurrent or persistent (24 hrs) or symptomatic diastolic BP > 110 mmHg, monotherapy is indicated | Delay until symptoms resolve and diastolic BP < 100 mmHg | 1^st^ event: no change |
|  |  | 2^nd^ event: subsequent dose reduced to 400 mg QD |
|  |  | 3^rd^ event: subsequent dose reduced to 200 mg QD |
|  |  | 4^th^ event: discontinue |
| Grade 3:  Recurrent or persistent (24 hrs) or symptomatic diastolic BP > 110 mmHg, requiring > 2 drugs, or more intensive therapy than previously | Delay until symptoms resolve and diastolic BP < 100 mmHg | 1^st^ event: subsequent dose reduced to 400 mg QD |
|  |  | 2^nd^ event: subsequent dose reduced to 200 mg QD |
|  |  | 3^rd^ event: discontinue |
| Grade 4:  Life-threatening consequences (e.g., hypertensive crisis) | Discontinue permanently (treat patient with anti-hypertensive agent immediately) |  |
